# What Is the Optimal Digoxin Level? Challenging Case of Fetal Atrial Flutter Treatment in a Monochorionic Diamniotic Twin

**DOI:** 10.3390/medicina59071198

**Published:** 2023-06-25

**Authors:** Soo Jung Kim, Hee Do Jeon, So-Yeon Shim, Yi-Seul Kim, Mi-Hye Park, Kyung A. Lee

**Affiliations:** 1 Department of Obstetrics and Gynecology, Ewha Womans University College of Medicine, Ewha Womans University Seoul Hospital, Seoul 07804, Republic of Korea; bossksj25@gmail.com (S.J.K.);; 2 Department of Cardiology, Loma Linda University Medical Center, Loma Linda, CA 92354, USA; heejeon@llu.edu; 3 Department of Pediatrics, Ewha Womans University College of Medicine, Ewha Womans University Seoul Hospital, Seoul 07804, Republic of Korea

**Keywords:** antiarrhythmic drug, digoxin, fetal atrial flutter, monochorionic diamniotic (MCDA) twin

## Abstract

*Background*: Atrial flutter is an infrequent yet potentially fatal arrhythmia. Digoxin is the preferred first-line treatment for fetal atrial flutter due to its efficacy and favorable safety profile. The optimal digoxin serum target level for neonatal atrial flutter management remains uncertain, with the standard target level ranging from 1.0 to 2.0 ng/mL due to potential toxicity concerns above this threshold. *Case Presentation*: We present a case of atrial flutter in a fetus within a monochorionic diamniotic (MCDA) twin pregnancy that was successfully managed using a higher-than-standard target level of digoxin. A 34-year-old nulliparous woman was referred to our institution at 31 + 3 weeks of gestation due to fetal distress in an MCDA twin pregnancy. Fetal echocardiography revealed a ventricular rate of 214 bpm in twin A, while twin B exhibited no abnormal findings. *Conclusions*: Our case highlights a distinct correlation between the serum digoxin level and its impact on atrial flutter. A higher target serum level of digoxin may be necessary to achieve sinus conversion due to the unique maternal and fetal circulatory characteristics in MCDA pregnancies.

## 1. Introduction

Fetal arrhythmia is a rare condition, affecting approximately 0.5% of all pregnancies. Atrial flutter, a potentially life-threatening arrhythmia, constitutes one-third of all fetal tachyarrhythmias and has been associated with a reported fetal mortality rate of up to 10% [1].

Digoxin is the preferred first-line treatment for fetal atrial flutter due to its proven effectiveness and favorable safety profile. The optimal target serum level remains uncertain; however, the widely accepted digoxin target level in clinical practice ranges from 1.0 to 2.0 ng/mL due to the concerns regarding potential toxicity at higher levels [2,3,4,5].

The treatment of fetal atrial flutter with digoxin in monochorionic diamniotic (MCDA) twin pregnancies can be challenging due to the pronounced maternal physiological adaptations during pregnancy and the reality that unpredictable placental perfusion may influence treatment outcomes [6,7]. Consequently, an individualized approach with a potentially higher target digoxin level might be necessary to achieve the desired therapeutic effect.

We present a case of atrial flutter in a fetus in an MCDA twin pregnancy that was successfully treated with a higher-than-usual target level of digoxin.

## 2. Case

A 34-year-old nulliparous woman was referred to our institution at 31 + 3 weeks of gestation due to fetal distress in an MCDA twin pregnancy. The result of transabdominal ultrasound assessment indicated fetal weights corresponding to 31 weeks of gestation and adequate levels of amniotic fluid. The placenta’s appearance on examination v9iia ultrasound was consistent with that seen in MCDA findings. 

**Figure 1:**
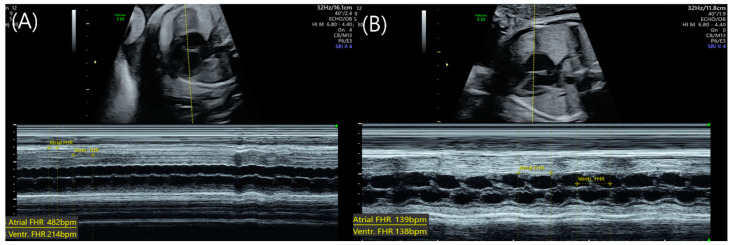
Atrial flutter in Twin A of an MCDA twin pregnancy. (**A**) M-mode image at 31 + 3 weeks of gestation, with calipers marking a ventricular rate of approximately 214 bpm. A 2:1 atrioventricular (AV) block is observed, as the atrial rate is approximately 482 bpm with two atrial beats per ventricular beat. (**B**) Following digoxin treatment, the fetal heart rhythm normalized to 138 bpm at 32 + 5 weeks of gestation.

Fetal echocardiography revealed a ventricular rate of 214 beats per minute (bpm) in twin A. Further evaluation using M-mode and Doppler ultrasound demonstrated an elevated atrial contraction rate, with approximately two atrial beats for each ventricular beat, leading to a diagnosis of atrial flutter with a 2:1 atrioventricular (AV) node conduction block (Figure 1A). Twin B exhibited no abnormal findings.

The mother conceived the MCDA twins through natural conception. Apart from hypothyroidism, she had no other medical or family history. Prior examinations at outside hospitals, including fetal ultrasound, prenatal screening, and maternal oral glucose tests, had all been unremarkable.

**Figure 2:**
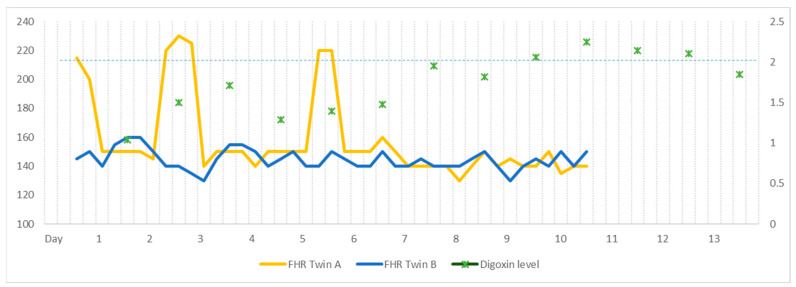
Twin fetal heart rate (FHR) and serum digoxin level (ng/mL) during treatment period (days).

Oral digoxin was administered to the mother with a loading dose of 0.5 mg, followed by 0.25 mg every 12 h, with a target serum level of 1–2 ng/mL. On day 2, twin A converted to a sinus rhythm at a serum level of 1.04 ng/mL; however, atrial flutter recurred at a serum level of 1.5 ng/mL on day 3. The digoxin dosage was increased by 0.25 mg every eight hours, and twin A transiently converted to sinus rhythm at a serum level of 1.71 ng/mL. Atrial flutter then returned on day 6 with a serum level of 1.39 ng/mL. The digoxin dose was further increased to 0.25 mg every six hours, and twin A converted to sinus rhythm the following day (Figure 1B). Between days 8 and 11, the digoxin level was maintained at a level between 1.82 and 2.25 ng/mL, and twin A remained at a normal sinus rhythm. On day 12, the digoxin level was 2.14 ng/mL, and the dose was reduced to 0.25 mg every eight hours. By day 17, the digoxin level was 1.85 ng/mL, with twin A remaining in sinus rhythm, and digoxin treatment was discontinued. Throughout this digoxin regimen, twin B maintained a normal sinus rhythm. The patient’s digoxin treatment regimen and corresponding serum levels are illustrated in Figure 2.

An elective cesarean section was conducted at 36 + 0 weeks of gestation without complications. Twin A, a male newborn, had a birth weight of 2650 g, a 1 min Apgar score of 8, and a 5 min Apgar score of 8. Twin B, also a male newborn had a birth weight of 2760 g, a 1 min Apgar score of 9, and a 5 min Apgar score of 10. All neonates exhibited appropriate birth weights, and the postpartum period was uneventful. Pathological examination of the placenta revealed a monochorionic diamniotic fused twin placenta.

**Figure 3:**
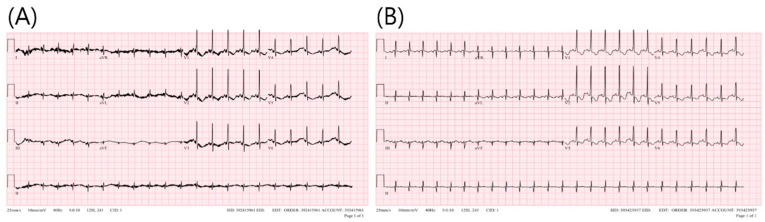
Pediatric electrocardiogram (ECG) of Twin A after birth (**A**) and on the 9th day (**B**).

Following birth, the newborn diagnosed with atrial flutter was admitted to the neonatal intensive care unit for further cardiac evaluation. Pediatric electrocardiography (ECG) demonstrated a sinus rhythm accompanied by non-specific ST and T wave changes (Figure 3A). On the 2nd day after birth, an abdominal ultrasound revealed the retention of retained meconium within the colonic loop, with other findings being unremarkable. On the 3rd day after birth, pediatric transthoracic echocardiography (TTE) identified a patent foramen ovale (PFO) measuring 3.5–4 mm, but the heart was otherwise structurally normal, with standard chamber size and ventricular function.

On the 9th day after birth, a follow-up pediatric ECG displayed normal sinus rhythm and non-specific ST and T wave changes (Figure 3B). The newborn was discharged on the 10th day without complications. The infant subsequently underwent follow-up as an outpatient with a pediatric cardiologist.

## 3. Discussion

Atrial flutter causes approximately 30% of fetal tachyarrhythmias. Untreated atrial flutter may cause low cardiac output and circulatory collapse due to uncontrolled tachycardia, which may lead potentially to fetal hydrops and even fetal death. Fortunately, with timely detection and treatment, the majority of neonatal atrial flutter shows a good prognosis [8].

Key differential diagnoses of fetal atrial flutter are sinus tachycardia, supraventricular tachycardia, atrial fibrillation and atrial ectopic tachycardia. Sinus tachycardia usually presents with HR > 200 bpm with 1:1 ventricular conduction. M-mode will demonstrate a regular atrial contraction followed by ventricular contraction with variable atrioventricular interval time depending on the speed of impulse conduction of atrioventricular node. Supra-ventricular tachycardia (SVT) involves a re-entrant pathway at the site of the atrioventricular node. SVT typically presents with a heart rate of 220–240 bpm with a monotonous fetal heart rate with a lack of atrial or ventricular rate variability. Ventricular repolarization occurs first, followed by retrograde activation of atrium. Therefore, M-mode will exhibit 1:1 ratio between atrial contraction and ventricular contraction, with the demonstration of ventricular contraction followed by atrial contraction. This is in contrast to atrial flutter with a 1:1 ratio in which atrial contraction always occurs before the ventricular contraction. Fetal ectopy is associated with approximately 1% of congenital cardiac defects. Atrial ectopic tachycardia arises from one or more atrial ectopic foci in the atrium. It is manifested via irregular atrial activity with ventricular rate ranging from 150–250 bpm.

Fetal atrial flutter is distinct from other arrhythmias in that it involves a macro-reentrant pathway that originates above the atrioventricular node, demonstrating a rapid and regular atrial activity. Fetal atrial flutter invariably presents with a constant atrial rate of >300 with variable atrioventricular conduction, leading to ventricular rate of either 150 bpm or 75 bpm depending on the degree of atrioventricular block. The atrioventricular conduction block has a 2:1 in 80% of cases and a 3:1 ratio in the remainder. In our case, the fetus demonstrated a regular atrial activity at rate of 482 with 2:1 ventricular conduction. Enhancing the refractoriness of the AV node may effectively control the ventricular rate and may consequently terminate the arrhythmia [9,10]. Fetal echocardiography with M-mode demonstrated that the relationship between atrial and ventricular contraction plays a crucial role when evaluating fetal arrhythmias. In addition to the M-mode, Doppler echocardiography with sample volumes measured at the left ventricular outflow tract, simultaneously measuring mitral inflow and forward aortic flow, can be used to demonstrate the relationship between the atrial and ventricular contraction (Table 1).

Digoxin, a low-molecular-weight drug, readily crosses the placenta and exhibits a rapid vago-mimetic effect on the fetal heart rate, rendering it an efficacious agent for treating fetal arrhythmias. The use of digoxin is particularly beneficial in managing unstable, incessant tachyarrhythmias, as it effectively decelerates AV node conduction while simultaneously exerting a positive inotropic effect on contractility [11].

A study by Zhou demonstrated that, following maternal intravenous digoxin therapy, fetal echocardiography showed improved hemodynamic profiles, including the Tei index, cardiovascular profile score, and umbilical artery resistance index, each of which correlated with the maternal digoxin levels [4].

Digoxin has a narrow therapeutic index that can produce severe adverse effects at supratherapeutic levels, necessitating close monitoring. Toxicity is more likely when digoxin levels exceed 2.0 ng/mL [12]. The optimal digoxin serum target level for treating neonatal atrial flutter remains uncertain, with various sources recommending target levels between 1.5 and 3.0 ng/mL. Jaeggie et al. reported that target digoxin level of 2–2.5 ng/mL resulted in a 21% sinus rhythm conversion rate in singleton pregnancies [13].

In contrast, Sridharan found that a target level of 2.0–3.0 ng/mL yielded a 51.7% conversion rate, while Miyoshi observed a 59% conversion rate at a target level of 1.5–2 ng/mL [14,15]. A 2014 AHA statement advocates the use of maternal intravenous digoxin as an initial therapy with a target range of 0.7–2.0 ng/mL [11]. However, this recommendation is grounded in expert consensus rather than randomized trials.

Our case study demonstrated the existence of a clear association between serum digoxin levels and the restoration of sinus rhythm in a fetus. The fetus experienced refractory atrial flutter when serum levels were below 1.5 ng/mL, while sinus rhythm was maintained within a 1.7–2.2 ng/mL range. This observation implies that higher target serum levels may be necessary in MCDA pregnancies.

Determining the optimal therapeutic digoxin level can be challenging in twin pregnancies. Twin pregnancies may require higher loading and maintenance doses to achieve target levels as hemodynamic adaptations, including plasma volume expansion and increased plasma-binding protein are more pronounced compared to singleton pregnancies [6].

The treatment of fetal atrial flutter in MCDA twin pregnancies presents further complexities. MCDA pregnancies are characterized by a single placenta shared between two separate vascular territories connected via vascular anastomoses, allowing for continuous transfusion between the twins. Depending on the size and location of these vascular anastomoses, fetuses may receive imbalanced perfusion, which may lead to unequal exposure to the maternal digoxin therapy [16].

In a study of fetal cardiac function in MCDA pregnancies, Torres et al. demonstrated that, in comparison to singleton pregnancies, fetal hearts in MCDA pregnancies exhibited a higher left myocardial performance index, as well as an elevated baseline cardiac output. These findings may not only suggest an increased circulating plasma volume, but also a potentially heightened baseline sympathetic tone [7].

In summary, considering the higher plasma volume, the potential presence of uneven placental perfusion between twins, and the increased baseline sympathetic tone, a higher target serum level may be necessary to achieve therapeutic effects in MCDA pregnancies.

Regarding the side effects of digoxin therapy, maternal administration has been deemed safe, with no reported teratogenicity, and maternal effects are often rare and mild [12]. In a case review of 10 instances of fetal arrhythmia treatment conducted by Chimenea et al. 3 out of 10 mothers experienced mild gastrointestinal symptoms as side effects. In all cases, digoxin levels were observed to be above 2 ng/mL, with symptoms resolved within 48 h. Other infrequent clinical toxicities associated with digoxin include vision changes and cognitive disturbances. Although toxicity is more likely when serum levels exceed 2.0 ng/mL, it is important to emphasize that digoxin serum levels do not always correlate with the severity of toxicity [3].

## 4. Conclusions

To the best of our knowledge, this represents the first case report delineating the successful treatment of fetal atrial flutter within the context of an MCDA pregnancy. Higher target digoxin serum levels may be required for sinus conversion, especially given the distinct maternal and fetal circulatory characteristics inherent to MCDA pregnancies. Further studies are warranted to ascertain the optimal therapeutic approach for addressing fetal atrial flutter in MCDA pregnancies.

## Figures and Tables

**Table 1:** Summary of characteristic findings of echocardiographic M-mode for each differential diagnosis of atrial flutter.

Tachycardia	Rhythm	Atrial Rate (Beats per Minute)	Atrioventricular Conduction	Ventricular Rate (Beats per Minutes)
Sinus tachycardia	Regular	180–200	1:01	180–200
Atrial flutter	Regular	350–500	1:1, 2:1, 3:1, 4:1	Variable depending on AV conduction
Supra-ventricular tachycardia	Regular	220–300	1:1 ventricular contraction followed by atrial contraction	220–300
Atrial ectopic tachycardia	Irregular	160–250	1:01	160–250

## Data Availability

The data presented in this study are available on request from the corresponding author. The data are not publicly available due to privacy and ethical concerns.
